# Neurofilament light chain levels predict encephalopathy and outcome in community‐acquired pneumonia

**DOI:** 10.1002/acn3.51711

**Published:** 2022-12-08

**Authors:** Ha‐Yeun Chung, Jonathan Wickel, Marcus Oswald, Justina Dargvainiene, Jan Rupp, Gernot Rohde, Martin Witzenrath, Frank Leypoldt, Rainer König, Mathias W. Pletz, Christian Geis, CAPNETZ Study Group

**Affiliations:** ^1^ Section of Translational Neuroimmunology, Department of Neurology Jena University Hospital Jena Germany; ^2^ Center for Sepsis Control and Care Jena University Hospital Jena Germany; ^3^ Systems Biology Research Group, Institute for Infectious Diseases and Infection Control (IIMK) Jena University Hospital Jena Germany; ^4^ Neuroimmunology, Institute of Clinical Chemistry and Department of Neurology, UKSH Kiel/Lübeck Kiel University Kiel Germany; ^5^ Department of Infectious Diseases and Microbiology University Hospital Schleswig‐Holstein Lübeck Germany; ^6^ CAPNETZ STIFTUNG Hannover Germany; ^7^ Biomedical Research in Endstage in Obstructive Lung Disease Hannover (BREATH) German Center for Lung Research (DZL) Hannover Germany; ^8^ Department of Respiratory Medicine, Medical Clinic I Frankfurt University Hospital, Goethe University Frankfurt Frankfurt/Main Germany; ^9^ Department of Infectious Diseases and Respiratory Medicine Charité – Universitätsmedizin Berlin Berlin Germany; ^10^ Institute of Infectious Diseases and Infection Control Jena University Hospital Jena Germany

## Abstract

**Objective:**

Serum neurofilament light chain (sNfL) is a biomarker for neuroaxonal damage and has been found to be elevated in several neurological diseases with neuronal destruction. New onset of confusion is a hallmark of severity in infections. The objective of this study was to determine whether sNfL levels are increased in patients with community‐acquired pneumonia (CAP) and if increased sNfL levels are associated with disease‐associated confusion or disease severity.

**Methods:**

In this observational study, sNfL levels were determined with single‐molecule array technology in CAP patients of the CAPNETZ cohort with validated CRB (confusion, respiratory rate, and blood pressure)‐65 score. We determined associations between log‐transformed sNfL concentrations, well‐defined clinical characteristics, and unfavorable outcome in multivariable analyses. Receiver operating characteristic (ROC) analysis was performed to assess the prediction accuracy of sNfL levels for confusion in CAP patients.

**Results:**

sNfL concentrations were evaluated in 150 CAP patients. Patients with confusion had higher sNfL levels as compared to non‐confusion patients of comparable overall disease severity. ROC analysis of sNfL and confusion provided an area under the curve (AUC) of 0.73 (95% CI 0.62–0.82). Log‐transformed sNfL levels were not associated with general disease severity. In a logistic regression analysis, log2‐sNfL was identified as a strong predictor for an unfavorable outcome.

**Interpretation:**

sNfL levels are specifically associated with confusion and not with pneumonia disease severity, thus reflecting a potential objective marker for encephalopathy in these patients. Furthermore, sNfL levels are also associated with unfavorable outcome in these patients and might help clinicians to identify patients at risk.

## Introduction

Encephalopathy caused by systemic inflammation affects up to 50% of patients resulting in delirium‐associated symptoms, such as confusion, agitation, or coma.^1^, ^2^ Infection‐associated confusion can be the first and only symptom of patients and is directly associated with in‐hospital mortality.^3^, ^4^, ^5^ Encephalopathy during the course of systemic inflammation (e.g., sepsis) might lead to long‐term neurocognitive deficits in survivors.^6^, ^7^, ^8^, ^9^

Community‐acquired pneumonia (CAP) is a common and serious disease condition and is the most frequent cause of community‐acquired sepsis. CAP is defined as an acute infection of the lung parenchyma which has been acquired outside a hospital.^10^ This is particularly important since CAP is characterized by a different spectrum of pathogens and therefore requires different empiric antibiotic therapy in comparison to hospital‐acquired pneumonia.^11^, ^12^ Patients with CAP show high variability in the disease course, which is dependent on the pathogen and patients' characteristics (e.g., age, comorbidities). Approximately 10% to 20% of hospitalized CAP patients require intensive care treatment and may ultimately suffer from multi‐organ failure during sepsis with high mortality rates.^13^, ^14^

The CRB‐65 – and its variants CURB and CURB‐65 – and the (q)SOFA score are widely used scores in CAP and sepsis. These scores are readily accessible to clinicians and are validated for the assessment of disease severity in emergency departments.^14^, ^15^, ^16^ The C(U)RB‐65 divides patients into low‐, moderate‐, or high‐risk groups for mortality based on clinical indicators: confusion (new onset); urea (>7 mmol/L); respiratory rate ≥ 30/min; blood pressure (systolic < 90 mmHg or diastolic ≤ 60 mmHg); age (≥65 years).^17^, ^18^, ^19^

Currently, there are no well‐established serum‐based laboratory biomarkers predicting confusion and encephalopathy in patients with CAP and other systemic inflammatory diseases. Neurofilaments are intracellular cytoskeletal proteins, which are abundant in neurons and are categorized according to their molecular weight into light, medium, and heavy chain types. Increased neurofilament light chain levels can be found in the cerebrospinal fluid (CSF) and also in periphery blood as a result of neuroaxonal damage regardless of underlying disease pathomechanisms. Serum neurofilament light chain (sNfL) is becoming a widely accepted marker for neuronal axonal damage in numerous neurological diseases, such as amyotrophic lateral sclerosis or multiple sclerosis.^20^, ^21^ Recent technological advances allow accurate measurement of low sNfL concentrations which directly relate to neuronal destruction in CNS disorders and strongly correlates with NfL concentration in CSF.^22^, ^23^ These advantages without the necessity of CSF analysis led to larger sNfL biomarker studies in several neurological disorders and the establishment of sNfL as a valid biomarker for neuronal damage.^24^, ^25^ Recent studies have characterized sNfL during systemic inflammation and provided the first evidence that sNfL may serve as a biomarker of neuronal injury.^26^, ^27^, ^28^

In this study, we aimed at investigating serum levels of sNfL in CAP patients with different disease states and we intended to assess the prognostic value of sNfL in CAP patients with associated confusion on long‐term outcomes. We hypothesized that sNfL levels are specifically associated with confusion rather than with the general disease severity in CAP patients and that sNfL levels allow a prognostic assessment for unfavorable outcome (e.g., death).

## Methods

### Study population

A total of 150 CAP patients from the CAPNETZ (Community‐Acquired Pneumonia Competence Network) cohort were included in the study. 150 CAP patients were divided into five groups: thirty age‐ and sex‐matched patients were selected for five disease severity states each identified by CRB scoring (confusion, respiratory rate, and blood pressure): CRB0, CRB1 without confusion, CRB1 with confusion, CRB2 without confusion, CRB2 with confusion. Patients with pre‐existing neurological diseases in the medical record were excluded to reduce the risk of bias of increased sNfL levels due to primary neuronal damage. Serum samples of all patients were provided by the CAPNETZ consortium. The CAPNETZ study (German Clinical Trials Register: DRKS00005274; see acknowledgment or www.capnetz.de for participating centers) is a prospective observational multicenter cohort study of CAP patients treated in the hospital or the outpatient setting. Samples were obtained from different geographical regions recruited by the CAPNETZ study group and were taken within 72 h after first contact with the recruiting hospital. CAPNETZ inclusion criteria were age ≥18 years, radiologically confirmed pneumonia, and at least one of the following clinical findings: cough, purulent sputum, fever, or focal chest sign on auscultation. Exclusion criteria were hospitalization during the 28 days preceding the study, immunosuppression, and active tuberculosis.^29^ All patients or their legal representatives provided written informed consent prior to enrolment in the study. All patients were followed up according to a standardized protocol for 180 days and all clinical parameters were stored in an electronic database. The study was approved by the local ethical committees of each participating center.

### Single‐molecule array neurofilament light measurements

NfL assay by Quanterix HD‐X was used to measure sNfL levels. All available serum samples were analyzed for concentrations of NfL in pg/mL using a single molecule array technique with a commercially available Neurofilament light kit (NF‐light) on a fully‐automated HD‐X platform. Biomarker concentrations were quantified using calibration curves with known standards from the NF‐light Kit, which were measured during each run. Assays were performed according to the manufacturer's protocols except for analyzing singlicates (due to the limited volume of serum samples) instead of duplicates after determining the very low coefficient of variance between the replicates in the validation runs.

In summary, we performed inter‐assay, intra‐assay comparisons, and precision measurements as well as inter‐laboratory comparisons of NfL measurements and identified an intra‐ and inter‐assay coefficient of variance below 10% in samples with low as well as high sNfL levels and excellent inter‐laboratory correlations (Pearson correlation coefficient 0.9986) as described elsewhere.^30^

### Statistical analyses

All data are presented as median and IQR (25th, 75th percentiles). sNfL levels were not normally distributed as tested by Shapiro–Wilk normality test. Outliers were excluded using the Grubbs test. sNfL concentrations were compared between groups using the Mann–Whitney U test or the Kruskal‐Wallis test. The ability of sNfL to predict confusion levels among these groups was assessed by estimations of the area under the curve (AUC) and their 95% CIs from receiver operating characteristic (ROC) analyses. Cutoff points for sNfL with diagnostic value were determined by likelihood ratio positive = sensitivity/(1 − specificity) from the described ROC curve and were derived to maximize sensitivity and specificity using the Youden index. For further analyses, sNfL levels were log‐transformed using the natural log to fulfill the normal distribution assumption. Correlation analysis was performed using the Pearson test with log‐transformed sNfL values.

Prior to linear and regression analysis sNfL, clinical characteristics and laboratory parameters were log‐transformed and internally standardized using *z*‐scores. Multivariable linear regression was used to analyze the association between log2‐sNfL and clinical characteristics and 95% CIs. Binary logistic regression was used to analyze the association between clinical and laboratory parameters (e.g., log2‐sNfL, leukocytes, hematocrit, CRB‐65) level and unfavorable outcome, providing odds ratios and 95% CIs. Calibration was assessed by the Hosmer‐Lemeshow test to determine goodness of fit. The unfavorable outcome was defined as the following events at the 14‐day follow‐up: death, ongoing hospitalization, and transfer to a rehabilitation clinic or nursing home. We used multiple imputations for missing data in the multivariable analysis and used all predictors together to impute missing values. For the multivariable model, missing values in the selected prognostic factors were imputed, subsequently combining the coefficients of each data set (*n* = 5) according to the Rubin rule. OriginPro 2019 was used to create box plots and modified by Inkscape 1.1. Statistical analysis was conducted using IBM SPSS Statistics data Editor (v.26).

## Results

A total number of 150 CAP patients were included in this study. Thirty patients in five severity groups either presenting clinical signs of confusion or no confusion were age‐ and sex‐matched in these groups: CRB0, CRB1 with no confusion, CRB1 with confusion (CRB1+confusion), CRB2 with no confusion, and CRB2 with confusion (CRB2+confusion). Patients' characteristics are presented in Table 1. The median age was 72 [IQR 64–82] years. The disease severity in the CRB2 in comparison to the CRB0 and CRB1 groups is reflected by an increased proportion of patients reporting dyspnea, an increased respiratory rate, and pulse. Furthermore, we found a higher incidence of heart disease in the CRB2 groups. However, we found no differences in other comorbidities, such as chronic kidney disease or chronic liver disease, which might affect sNfL levels independent from infection.

**Table 1:** Baseline and clinical characteristics of CAP patients with and without confusion.

	CRB0	CRB1	CRB1+confusion	CRB2	CRB2+confusion
Confusion (%)	0	0	100	0	100
Median age in years (IQR)	72 (64/81)	71 (64/83)	72 (64/82)	71 (64/83)	72 (64/81)
Female sex	15/30	15/30	15/30	15/30	15/30
Fever (>38°C)	13/30	14/30	17/30	24/30	23/40
Smoking	5/30	7/30	5/30	10/30	12/30
Tumor	2/30	2/30	1/30	4/30	2/30
Chronic respiratory disease	7/30	7/30	12/30	13/30	10/30
Heart disease	13/30	16/30	14/30	19/30	19/30
Chronic kidney disease	1/30	0/30	1/30	4/30	1/30
Chronic liver disease	2/30	3/30	4/30	7/30	3/30
Diabetes mellitus	4/30	6/30	4/30	7/30	9/30
Infiltrate	30/30	30/30	30/30	30/30	30/30
Multilobular infiltrate	7/30	7/30	3/30	9/30	9/30
Pleural effusion	5/30	4/30	4/30	9/30	5/30
Median systolic blood pressure in mmHg (IQR)	140 (115/150)	120 (110/130)	130 (120/140)	110 (100/120)	120 (103/135)
Median diastolic blood pressure in mmHg (IQR)	80 (70/125)	70 (60/96)	80 (80/90)	60 (60/60)	70 (60/105)
Median pulse	80 (73/90)	87 (80/104)	91 (80/108)	98 (80/117)	100 (100/120)
Median respiratory rate	18 (16/20)	22 (18/30)	22 (20/25)	34 (31/36)	25 (20/30)
Median temperature	37.2 (37.0/38.0)	37.4 (36.9/38.6)	37.9 (37.0/38.8)	38.0 (37.0/38.9)	38.6 (36.8/39.1)
Dyspnea	22/30	21/30	23/30	27/30	27/30
Pre‐antibiotic treatment (<4 weeks)	7/30	5/30	6/30	8/30	5/30
Median antibiotic treatment in days (IQR)	9 (7/10)	8 (7/10)	10 (7/14)	11 (9/14)	9 (7/15)
Need for oxygen	15/30	23/30	26/30	23/30	27/30
Median leukocytes in 10^9^/L (IQR)	12.3 (8.7/16.7)	11.4 (9.0/20.1)	10.3 (7.2/14.8)	12.8 (11.0/16.2)	14.6 (12.8/18.8)
Median hemoglobin in mmol/L (IQR)	8.0 (7.7/9.0)	8.0 (7.3/8.7)	8.1 (7.4/9.4)	8.5 (7.7/8.9)	7.4 (6.1/8.0)
Median hematocrit in % (IQR)	39 (37/43)	39 (34/41)	38 (35/44)	40 (37/43)	36 (32/40)
Median thrombocytes in 10^9^/L (IQR)	230 (188/264)	231 (170/287)	227 (161/284)	207 (158/237)	198 (165/294)
Median pH (IQR)	7.4 (7.4/7.5)	7.4 (7.4/7.5)	7.4 (7.4/7.5)	7.5 (7.4/7.5)	7.5 (7.4/7.5)
Median po_2_ in mmHg (IQR)	60 (53/71)	71 (59/86)	61 (57/83)	54 (48/62)	58 (47/73)
Median pco_2_ in mmHg (IQR)	32 (28/39)	34 (30/41)	40 (35/48)	34 (32/38)	34 (30/42)
Median o_2_ saturation in % (IQR)	93 (90/95)	92 (90/96)	91 (88/96)	92 (88/95)	93 (90/95)
Median CrP in mg/L (IQR)	146 (19/249)	89 (28/185)	41 (14/159)	129 (72/273)	196 (64/246)
Median urea in mg/dL (IQR)	5.0 (4.2/7.9)	6.9 (4.2/11.8)	7.3 (5.5/10.5)	8.0 (6.7/11.2)	8.0 (6.5/13.2)
Median glucose in mmol/L (IQR)	6.6 (6/8.6)	7.4 (6/8.7)	7.5 (6.1/8.9)	6.9 (5.9/9.3)	8.5 (5.8/11.3)
Median sodium in mmol/L (IQR)	137 (133/140)	137 (136/140)	137 (133/139)	134 (132/138)	132 (137/139)

CRB: C, confusion; R, respiratory rate; B, blood pressure.

### Serum NfL levels are associated with confusion but not with disease severity

**Figure 1:**
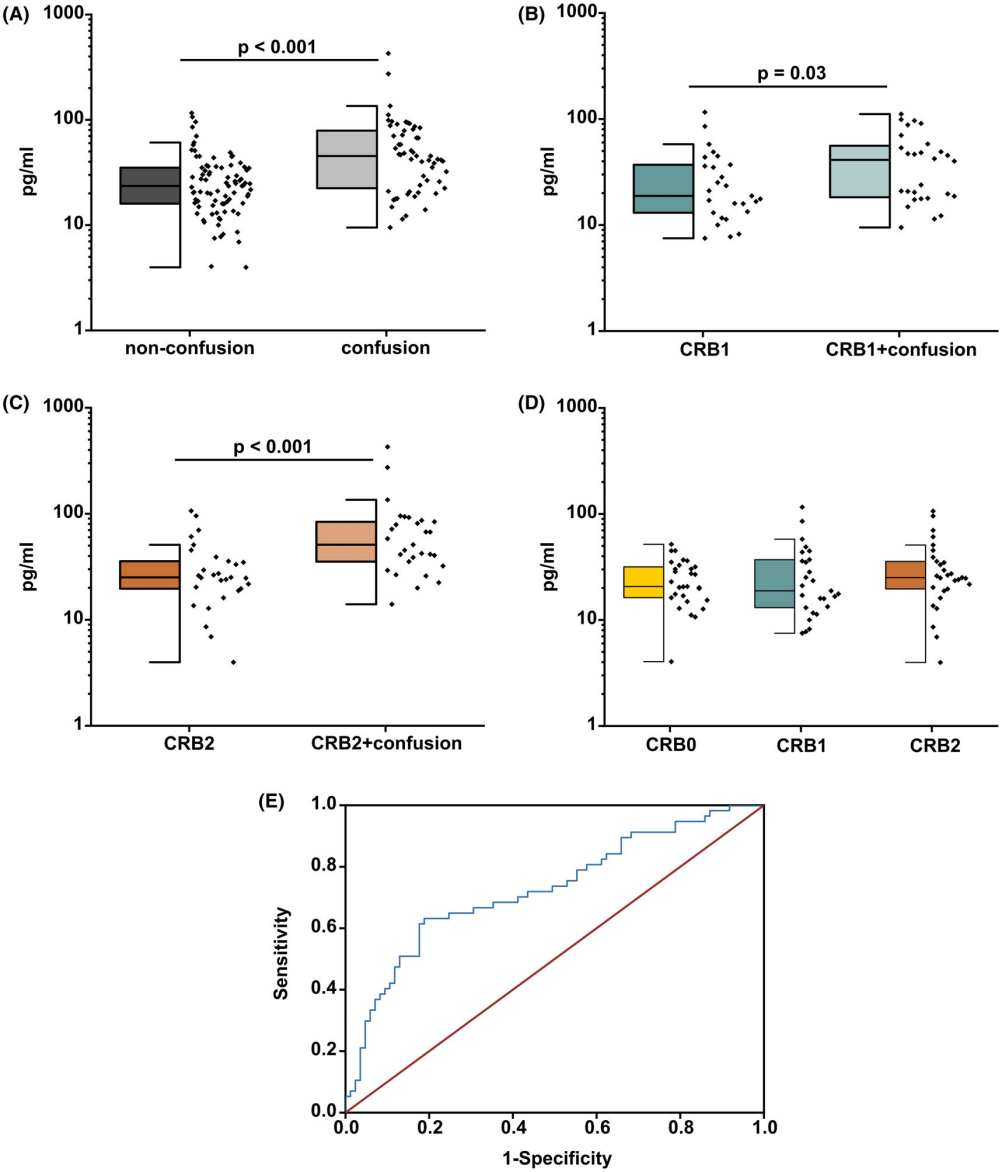
Serum NfL levels in different CAP severity groups with and without confusion. (A) Comparison of serum NfL levels in 58 patients with confusion and 87 patients without confusion shows significantly higher serum NfL levels in CAP patients with confusion (Mann–Whitney‐*U* test; *p* < 0.001). (B,C) Higher serum NfL levels in subgroups of 29 CRB1 patients compared to 29 CRB1+confusion patients (Mann–Whitney‐*U* test; *p* = 0.0302) and 29 CRB2 patients compared to 29 CRB2+confusion patients (Mann–Whitney‐*U* test; *p* < 0.001). (D) No differences were observed when comparing groups with different CAP disease severity: CRB0, CRB1, and CRB2 (Kruskal‐Wallis ANOVA; *p* = 0.488). Boxes represent the median and interquartile range [IQR] and whiskers the extreme value within 1.5 × IQR above or below median. (E) ROC Curve showing serum NfL levels predicting confusion (AUC: 0.73; 95% CI 0.62–0.82). CRB: C, confusion; R, respiratory rate; B, blood pressure.

The overall median of sNfL levels was 27.88 pg/mL (IQR 17.69–47.08 pg/mL). Direct comparison of sNfL levels in all CAP patient groups without confusion (CRB0, CRB1, CRB2) to CAP patient groups with confusion (CRB1+confusion, CRB2+confusion) showed higher sNfL concentrations in the groups with confusion (median 23.55 pg/mL [IQR 15.93–35.42 pg/mL] vs. 45.35 pg/mL [IQR 21.68–80.01 pg/mL]; *p* < 0.001) (Fig. 1A). To further evaluate whether pneumonia severity was associated to sNfL levels we compared to age‐ and sex‐matched groups with different disease severity and occurrence of confusion. As shown in Fig. 1B,C we found higher sNfL concentrations in CRB1+confusion and CRB2+confusion compared to their matched patient cohort with similar disease severity (CRB1: median 18.82 pg/mL [IQR 13.06–37.01 pg/mL] vs. CRB1+confusion: 41.17 pg/mL [IQR 18.1–57.09 pg/mL]; *p* = 0.031; CRB2: median 25.09 pg/mL [IQR 19.21–37.39 pg/mL] vs. CRB2+confusion: 51.02 pg/mL [IQR 33.75–85.25 pg/mL]; *p* < 0.001). Importantly, we found no differences in sNfL levels comparing different severity groups (CRB0 vs. CRB1 vs. CRB2) without signs of confusion indicating that increased sNfL levels are specifically attributed to confusion rather than overall disease severity in CAP (Fig. 1D).

We further investigated whether clinical characteristics and laboratory parameters were associated with increased sNfL levels. Therefore, we log‐transformed and standardized all clinical characteristics and sNfL levels prior to further analyses. Performing a multivariable linear regression analysis with patients' characteristics as an independent variable and log2‐sNfL as a dependent variable, we observed that higher log2‐sNfL values were closely associated with confusion (regression coefficient: 0.38, 95% CI 0.19–0.56, *p* < 0.001). Furthermore, age (regression coefficient: 0.21, 95% CI 0.01–0.41, *p* = 0.04), chronic kidney disease (regression coefficient: 0.26, 95% CI 0.07–0.46, *p* = 0.008), and lower levels of hematocrit (regression coefficient: −0.27, 95% CI −0.43 to −0.12, *p* = 0.001) were also associated to higher levels of log2‐sNfL. Again, no association between log2‐sNfL and disease severity score (CRB‐65) was found (Table 2).

**Table 2:** Factors associated with increased log‐transformed sNfL levels in CAP.

	β‐coefficient	Lower bound 95% CI	Upper bound 95% CI	*p*‐value
Confusion^3^	0.38	0.19	0.56	**<0.001** *
Age^2^	0.21	0.01	0.41	**0.040** *
Chronic airway disease^3^	0.04	‐0.12	0.21	0.607
Chronic heart failure^3^	0.04	−0.15	0.23	0.715
Chronic kidney disease^3^	0.26	0.07	0.46	**0.008** *
Body temperature^2^	−0.43	−0.22	0.14	0.641
Leukocytes^2^	−0.03	−0.21	0.15	0.716
Hematocrit^2^	−0.27	−0.43	−0.12	**0.001** *
po_2_ ^2^	0.07	−0.13	0.27	0.476
pco_2_ ^2^	−0.06	−0.24	0.14	0.493
CRB‐65^1^ ^,^ ^2^	0.16	−0.03	0.36	0.101
Sodium^2^	0.02	−0.15	0.19	0.787
Glucose^2^	−0.04	−0.25	0.17	0.719

^1^ CRB‐65: C, confusion; R, respiratory rate; B, blood pressure; 65, age > 65.

^2^ Log‐transformed and standardized (z‐score).

^3^ Standardized (*z*‐score).

* Statistically significant values have been bolded.

The area under the curve (AUC) for predicting confusion in CAP was 0.73 (95% CI 0.62–0.82). The optimal cutoff point in sNfL concentration according to the Youden index was 38 pg/mL (sensitivity 63% and specificity 81%) (Fig. 1E). As expected, we found a moderate correlation between log2‐sNfL and age (*r* = 0.56, *p* < 0.001) in the patient cohort showing no signs of confusion. However, we found a weak correlation between log2‐sNfL and age in the patient cohort showing signs of confusion (*r* = 0.27, *p* = 0.04) indicating that the effect of confusion alleviates the effect of age.

### Serum NfL is increased in CAP patients with unfavorable outcome

**Figure 2:**
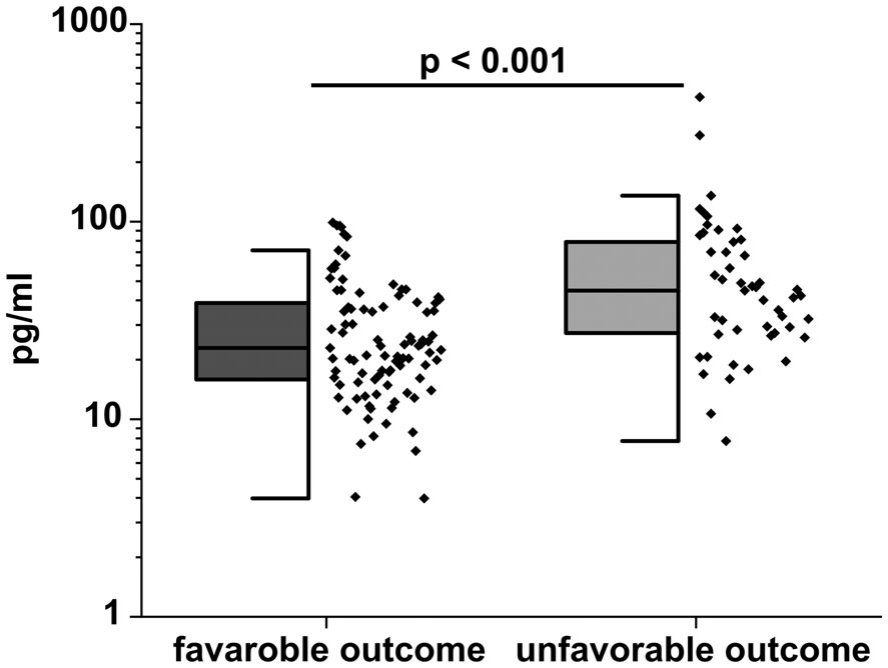
Comparison of serum NfL levels in 93 patients with favorable outcome and 49 patients with unfavorable outcome shows significantly higher serum NfL levels in CAP patients with unfavorable outcome (Mann–Whitney‐*U* test; *p* < 0.001).

Next, we were interested in whether sNfL levels were associated with unfavorable outcome. When directly comparing sNfL levels of patients with the unfavorable outcome as defined by death, ongoing hospitalization, and transfer to a rehabilitation clinic or nursing home on day 14 following hospitalization, we observed higher levels in these patients as compared to patients, who were discharged home in a period of 14 days (median 22.96 pg/mL [IQR 15.62–38.91 pg/mL] vs. 44.74 pg/mL [IQR 27.12–80.01 pg/mL]; *p* < 0.001) (Fig. 2).

In a logistic regression analysis, we found that only log2‐sNfL levels on admission (odds ratio (OR) = 2.68, 95% confidence interval (CI) 1.39–5.15; *p* = 0.003) and log2‐pco_2_ on admission (odds ratio (OR) = 2.74, 95% confidence interval (CI) 1.26–5.96; *p* = 0.013) independently predicting unfavorable outcome in CAP patients (Table 3). No association between unfavorable outcome and disease severity (CRB‐65 score) or other laboratory parameters (e.g., leukocytes, C‐reactive protein, hemoglobin) was found (Table 3). As expected, further analysis revealed an association between CRB‐65 score and death.

**Table 3:** Factors associated with an unfavorable outcome in CAP.

	Multivariable OR unfavaroble outcome (CI 95%)	*p*‐value multivariable analysis
sNfL^2^	2.68 (1.39–5.15)	**0.003** *
Leukocytes^2^	1.51 (0.77–2.96)	0.227
Hemoglobin^2^	0.98 (0.59–1.65)	0.947
Thrombocytes^2^	0.62 (0.35–1.11)	0.107
po_2_ ^2^	0.83 (0.43–1.60)	0.553
pco_2_ ^2^	2.74 (1.26–5.96)	**0.013** *
C‐reactive protein^2^	0.95 (0–54‐1.67)	0.847
Sodium^2^	1.09 (0.69–1.72)	0.706
CRB‐65 score^1^ ^,^ ^2^	1.46 (0.85–2.50)	0.173

^1^ CRB‐65: C, confusion; R, respiratory rate, B, blood pressure, 65, age > 65.

^2^ Log‐transformed and standardized (*z*‐score).

* Statistically significant values have been bolded.

## Discussion

Our study demonstrates that sNfL levels are specifically increased in CAP patients presenting with confusion, whereas sNfL levels were not associated with overall disease severity. Yet, we found that sNfL levels in CAP patients were the strongest independent predictor for unfavorable outcomes including ongoing hospitalization, transfer to a rehabilitation clinic or nursing home, or death.

In the last years, sNfL has been established as a biomarker of neuronal injury as well as a prognostic marker in numerous neurological diseases with neurodegeneration.^20^, ^21^ Moreover, recent studies evaluated its diagnostic potential also in the case of systemic inflammation regarding the prediction of an unfavorable outcome, such as death, need for mechanical ventilation or intensive care treatment.^26^, ^27^, ^28^ As for now, sNfL levels are suggested to be associated with disease severity and encephalopathy in several infectious diseases, such as sepsis in general or pathogen‐specific infectious diseases.^27^, ^28^, ^31^, ^32^ However, in this study we show that sNfL levels are more likely associated with the occurrence of encephalopathy (delirium or confusion) in the course of infection rather than with disease severity itself. This is in line with previous studies showing an increase in plasma NfL levels in patients with post‐operative delirium as a form of encephalopathy.^33^, ^34^ We assume that the increase in sNfL levels in infectious diseases is more likely to be attributed to an increased occurrence of encephalopathy in more severe stages of infection. Moreover, sNfL levels might be also influenced by the duration of symptoms, especially the length of delirium and/or confusion. Therefore, NfL levels might be underestimated in blood samples when symptoms of pneumonia developed acutely within less than 3 days. As already shown in many other studies, we also observed an association of sNfL levels with chronic kidney disease.^35^, ^36^ In studies investigating sNfL concentration in severe infections it is important to consider that sNfL levels are influenced by kidney function. Chronic kidney disease was evenly distributed across all groups (Table 1). In addition, we compared renal retention parameters and found no differences in urea levels comparing all groups (Table 1).

Interestingly, we observed that the patient cohort with CRB1c (showing only confusion) had higher sNfL levels than patients in the CRB1 group with no confusion presenting with affected respiration or blood pressure per definition. These results again underline the attribution of sNfL levels to confusion and not disease severity. Classification of encephalopathy in scores such as qSOFA or SOFA is still limited to the Glasgow coma scale.^16^, ^19^ However, these scores still lack information about confusion or delirium. Our data provide evidence that an increase in sNfL levels and confusion is associated with unfavorable outcome and should therefore be considered in the clinical evaluation and included in acute disease scores. Delayed or missed scoring of confusion or delirium might therefore lead to the conclusion that the brain is unaffected during acute disease stages. It is known that confusion or delirium are independent predictors for an unfavorable outcome in infectious diseases,^27^, ^37^, ^38^ thus supporting the importance of timely detection and objective and precise diagnosis of early encephalopathy. Our results may be interpreted that sNfL could be implemented in CAP patients as a biomarker to objectify confusion and delirium in infection‐associated severity scores, which is an independent predictor of unfavorable outcomes. Using a biomarker might help clinicians in the daily routine of busy emergency departments and further identify delirium subtypes, which are clinically more challenging to determine, such as hypoactive delirium.^39^, ^40^ Early identification of patients with confusion or delirium may reduce the risk for later development of long‐term cognitive impairment.^8^, ^9^, ^41^

Several considerations and limitations should be noted in our study. Since this study is designed from the multicenter observational CAPNETZ cohort, we cannot exclude residual confounding factors by unmeasured parameters, which is a general limitation in observational study cohorts. In addition, we defined the occurrence of encephalopathy in CAP patients with the clinical scoring of confusion during the assessment of the CRB‐65 score by the examining clinicians. We cannot differentiate between delirium and other presentation of encephalopathy, e.g., reduced consciousness, agitation, or anxiety. Therefore, future studies are needed to confirm our findings of sNfL increase in CAP‐induced encephalopathy for example with standardized delirium scores (e.g., CAM‐ICU). It is also important to note that we here provide evidence for CAP patients and not for patients with hospital‐acquired pneumonia which has a different pathogenic spectrum. Furthermore, from our data, we cannot generalize our conclusions to all patients with systemic inflammation and different infectious foci. However, since the lung is the most common focus of acquired sepsis, our results are likely representative of a high number of patients with systemic inflammation. In addition, it is noteworthy that the diagnostic accuracy of sNfL concentrations for predicting unfavorable outcome on the individual patient level is limited. Although sNfL levels are higher in the total cohort of patients with an unfavorable outcome, the data distribution largely overlaps, thus making it difficult to define a pathologic cutoff at the individual patient level. Last, as stated in the method section, we excluded all patients with preexisting neurological diseases in the medical history to reduce the risk of bias of primary neuronal damage. Since neurological conditions usually associated with increased sNf levels, such as Alzheimer's disease, have a long pre‐ and subclinical phase, this confounding factor cannot be ruled out completely. Furthermore, we cannot fully exclude reverse causation, e.g., that such disease states may also predispose to confusion during CAP.

The current study has an important practical implication. We here demonstrate that sNfL is a potential biomarker of confusion and encephalopathy in CAP patients indicating its possible use to support current infection‐associated disease severity scores (e.g., CRB‐65 or SOFA score). Furthermore, sNfL might help to identify patients at risk for unfavorable outcome, particularly for transfer into long‐term care facilities, already in the emergency room.

## Author Contributions

H.‐Y.C., J.W., C.G., R.K., M.O., and M.P. contributed to the conception and study design. Acquisition and analysis of data: H.‐Y.C., J.W., C.G., M.P., J.D., F.L., M.W., J.R., and G.R. Drafting a significant portion of the manuscript or figures: H.‐Y.C. and C.G.

## Conflict of Interest

The authors declare no competing financial interests.
